# Hands Deformity in a Patient with IgA Vasculitis

**DOI:** 10.31138/mjr.33.2.259

**Published:** 2022-06-30

**Authors:** Konstantinos Ntelis, George Iliopoulos, George Giannopoulos

**Affiliations:** 1 Private Practice, Kalamata, Greece,; 2 Division of Rheumatology, Department of Internal Medicine, Patras, University Hospital, University of Patras Medical School, Patras, Greece,; 3 Rheumatology Unit, Alexandra General Hospital, Faculty of Medicine, National and Kapodistrian University of Athens, Athens, Greece

**Keywords:** IgA vasculitis, Henoch–Schönlein purpura, Jaccoud’s arthropathy

## Abstract

74-year-old female patient with IgA vasculitis was referred for rheumatic evaluation due to arthritic complaints and hand deformities. Physical examination revealed reversible Jaccoud’s arthropathy in both hands, with swan-neck type deformities, while no erosions were present in the X-Ray. Jaccoud’s arthropathy is mainly observed can be present in patients with in Rheumatic Fever, Systemic Lupus Erythematosus, and Sjogren’s syndrome. The absence of erosions distinguishes this entity from rheumatoid arthritis. There is no specific treatment other than the treatment of the underlying disease.

## CASE PRESENTATION

A 74-year-old female patient diagnosed with IgA vasculitis (formerly known as Henoch–Schönlein purpura) was referred by her general practitioner for a rheumatologic evaluation. The diagnosis has been made 12 years ago and was based upon her typical clinical manifestations (purpura, gastrointestinal bleeding, glomerulonephritis) and the typical findings in kidney biopsy (dense deposits of IgA immunocomplexes and C3 complement).^1^ The patient had a chronic disease with frequent flares and was under chronic immunosuppressive treatment with steroids and azathioprine by her attending nephrologists. Arthritic complaints were minimal and she has never been referred to a rheumatologist.

**Figure 1.**
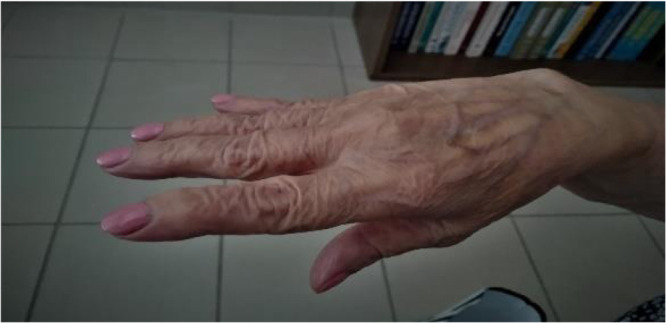
Arthritis of the right hand with ulnar deviation, swan-neck type deformities in the index and middle finger.

Main laboratory findings included anaemia (Haematocrit 33%, Hemoglobulin 10.9 gr/dl), elevated serum creatinine (1.72 gr/dl), haematuria (100 red cells per high power field), ANA(−), ANCA(−), RF(−) and anti-CCP(−) . Physical examination revealed arthritis in both hands and chronic swan-neck type deformities. The deformities were reversible. Radiographic findings included ulnar deviation, sublaxation of the 1^st^ MCP joints in both hands and absence of joint erosions. This clinical image is typical of Jaccoud’s arthropathy.

**Figure 2.**
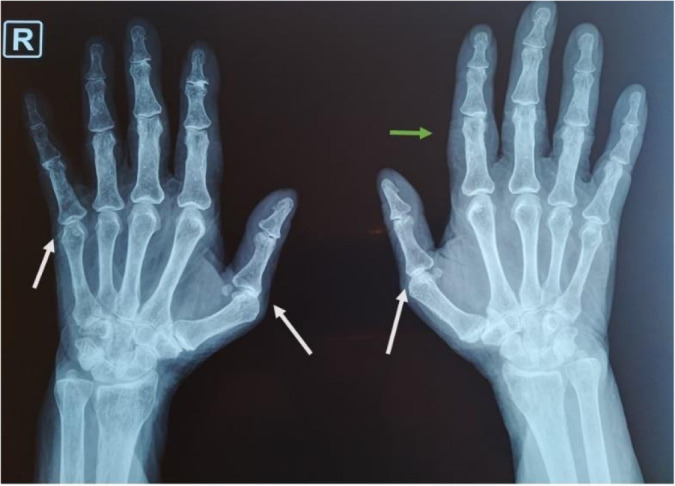
X-Ray shows ulnar deviation of both hands, subluxations of the 1st and 5th MCP joints (white arrows), soft tissue enlargement (green arrow) and absence of bone erosions.

Jaccoud’s arthropathy is mainly observed in Rheumatic Fever, Systemic Lupus Erythematosus and Sjogren’s syndrome but has been associated with several clinical entities.^2^ It is characterised by reversible deformities, usually affecting the MCP and MTP joints. The pathogenesis of the arthropathy is mostly attributed to capsule fibrosis and relaxation of tendons and ligaments. The most characteristic finding in X-rays is the absence of joint erosions, despite the presence of ulnar deviation and swan-neck deformities. This is the major clue for the differential diagnosis between Jaccoud’s arthropathy and rheumatoid arthritis. Treatment is mainly conservative and focuses on the underlying disease. To our knowledge this is the first case of Jaccoud’s arthropathy associated with IgA vasculitis in literature.

## ABBREVIATIONS

ANA: Antinuclear antibodies
ANCA: Anti-neutrophil cytoplasmic antibodies
Anti-CCP: Anti-cyclic citrullinated peptide antibodies
IgA: Immunoglobulin A
MCP: Metacarpophalangeal joints
MTP: Metatarsophalangeal joints
RF: Rheumatoid Factor
